# The Impact of Non-AKI eGFR Variability on CKD Progression in Individuals With Type 2 Diabetes and Preserved Kidney Function

**DOI:** 10.1016/j.ekir.2026.106667

**Published:** 2026-06-18

**Authors:** Simona Hapca, Qinbo Yang, Sheyu Li, Stuart J. McGurnaghan, Luke A.K. Blackbourn, Ewan R. Pearson, Helen M. Colhoun, Samira Bell

**Affiliations:** 1 Division of Computing Science and Mathematics, University of Stirling, Scotland, UK; 2 Department of Nephrology, West China Hospital, Sichuan University, Chengdu, China; 3 Department of Endocrinology and Metabolism, West China Hospital, Sichuan University, Chengdu, China; 4 Diabetes Informatics & Epidemiology Unit, MRC Institute of Genetics and Cancer, The University of Edinburgh, Edinburgh, UK; 5 Division of Molecular & Clinical Medicine, School of Medicine, University of Dundee, Dundee, UK; 6 College of Medicine and Veterinary Medicine, Usher Institute, The University of Edinburgh, Edinburgh, UK; 7 Division of Population Health and Genomics, School of Medicine, University of Dundee, Dundee, UK; 8 Division of Population and Behavioral Science, School of Medicine, University of St Andrews, North Haugh, St Andrews, UK

**Keywords:** GFR variability, chronic kidney disease, diabetes

## Abstract

**Introduction:**

Variability in estimated glomerular filtration rate (eGFR) has been associated with increased risks of mortality and chronic kidney disease (CKD) progression in people with type 2 diabetes mellitus (T2DM) and impaired kidney function. However, its significance in individuals with preserved kidney function remains unclear.

**Methods:**

In this nationwide retrospective population-based study of individuals with T2DM, eGFR variability was calculated by fitting a linear regression model to longitudinal data to estimate both the individual eGFR slope over the 5-year period as well as the variability in model residuals provided by the SD of the model residuals using longitudinal serum creatinine (SCr) measurements obtained during the first 5 years after diagnosis. Cox proportional hazards models were then applied to assess the association between eGFR variability and progression to stage G3b CKD among participants with preserved kidney function.

**Results:**

This study included 98,322 participants who had an eGFR > 60 ml/min per 1.73 m^2^ at diagnosis, remained alive with an eGFR > 60 ml/min per 1.73 m^2^ 5 years after diagnosis, and were subsequently followed for a mean of 5.1 years. Greater eGFR variability was associated with an increased risk of progression to stage G3b CKD— hazard ratios (HRs) for the second, third, and fourth quartiles of variability versus the first quartile were 1.56 (95% confidence interval [CI]: 1.38–1.75), 1.85 (95% CI: 1.65–2.08), and 2.56 (95% CI: 2.29–2.86), respectively. This association persisted after adjustment for multiple variables—HR: 1.57; 95% CI: 1.40–1.77 for the fourth quartiles of variability versus the first quartile.

**Conclusion:**

eGFR variability in the absence of acute kidney injury (AKI) is associated with CKD progression in individuals with T2DM and preserved kidney function.

## Graphical abstract

**Figure:**
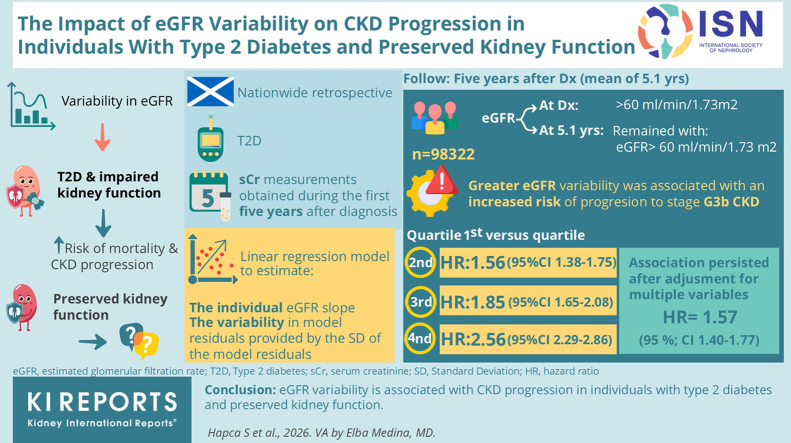

CKD is a major global public health challenge affecting approximately 10% to 15% of adults worldwide.^1^ Among patients with CKD, the burden of comorbidities and associated complications significantly impairs health-related quality of life and increases mortality.^2^ The global rise in CKD prevalence parallels the increasing incidence of T2DM, a major risk factor for CKD.^3^^,^^4^ Therefore, early risk stratification among individuals with diabetes is essential for primary prevention of CKD.

Both a decline in eGFR and the presence of albuminuria are well-established, independent predictors of adverse clinical outcomes in individuals with diabetes.^5^, ^6^, ^7^ However, a notable proportion of individuals with diabetes experience early eGFR decline and rapid progression to CKD even in the absence of microalbuminuria or macroalbuminuria.^8^, ^9^, ^10^ In these people without overt diabetic nephropathy, a single assessment of eGFR and albuminuria is frequently insufficient to accurately capture the risk of future kidney function decline.^11^, ^12^, ^13^

Emerging evidence suggests that longitudinal variability in clinical and biochemical parameters, especially in blood pressure, and hemoglobin A1c (HbA1c), are linked to increased risks of various adverse outcomes.^14^, ^15^, ^16^, ^17^, ^18^, ^19^ Variations in kidney function may indicate underlying kidney disorders, such as reduced renal reserve and nephron loss, but can also be influenced by external factors like dehydration, severe diarrhea, or congestive heart failure. Many of these factors are closely tied to CKD progression and associated with increased mortality.^20^^,^^21^ Although the predictors and prognostic value of eGFR variability have been examined in patients with moderate-to-advanced CKD,^20^, ^21^, ^22^, ^23^ its role in individuals with diabetes and preserved kidney function remains uncertain.^24^^,^^25^

The aim of this study is to evaluate the prognostic significance of eGFR variability in individuals with T2DM and preserved kidney function and its association with mortality and subsequent development and progression of CKD.

## Methods

### Study Design, Inclusion and Exclusion Criteria

This national retrospective cohort included all adults aged > 35 years resident in Scotland with a diagnosis of diabetes from January 2005 to December 2021 with an eGFR > 60 ml/min per 1.73 m^2^ at diagnosis. Individuals were followed up for 5 years from diagnosis, with those who died within 5 years from diagnosis excluded from the study. In addition, those who developed CKD (eGFR< 60 ml/min per 1.73 m^2^) within 5 years from diagnosis were also excluded from the study to ensure that the cohort was formed of people with preserved kidney function. People with less than 5 SCr measures (measured on 5 separate days) within the 5 years of initial follow-up and no SCr measure in the year preceding the 5 years from diagnosis were also excluded.

### Datasets and Variables

The previously described Scottish Diabetes Research Network (SDRN)–National Diabetes Study formed the study cohort.^26^ It is estimated that > 99% of those diagnosed with diabetes in Scotland are included within this cohort with linkage to multiple routine care databases. As a result, anonymized healthcare routine data of unselected people with diabetes from Scotland provided by the Scottish Care Information – Diabetes national register, was linked to demography, mortality, hospital admission records, laboratory biochemistry, and prescribing through the unique National Health Service (NHS) Scotland Community Health Index. The Scottish Care Information-Diabetes Collaboration data contains information on diabetes including type and date of diabetes diagnosis, and regular screening measures for diabetes progression such as HbA1c, systolic and diastolic blood pressure, and body mass index (BMI). Both primary and secondary care SCr values were obtained from the laboratory biochemistry data. The Scottish Renal Registry was used to identify patients receiving kidney replacement therapy and date of therapy initiation.^19^ The Scottish Morbidity Records 01 for hospital admission was used to evaluate patient comorbidities including coronary artery disease, congestive heart failure, peripheral vascular disease, and cerebrovascular disease based on ICD-9 and ICD-10 codes reported at discharge.^27^ The community prescribing data were used to gain information on the dates patients were prescribed angiotensin converting enzyme inhibitors, angiotensin receptor blockers, and sodium-glucose cotransporter 2 inhibitors before T2DM diagnosis or during the 5 years of follow-up from diagnosis. The demographics dataset was used to determine participant sex and date of birth, which was used to calculate age at diabetes diagnosis. The National Records for Scotland dataset was used to obtain the date of death. Albuminuria measurements were not included in the primary analysis because of substantial missingness and irregular testing during the exposure window, which precluded reliable incorporation into the models.

The Community Health Index number (the NHS Scotland unique patient identifier) was used to link Scottish Care Information-Diabetes Collaboration to biochemistry, Scottish Morbidity Records 01, community dispensed prescribing, demography, and National Records for Scotland datasets.

Data access and linkage was facilitated by the SDRN with analysis of the anonymized patient data being carried out on a trusted research environment. Access to the Scottish NHS diabetes data sources was granted to the SDRN epidemiology research purposes by approval from the Public Benefit and Privacy Panel for Health and Social Care (reference 1617–0147).^26^

### eGFR and CKD Stages

**Figure 1:**
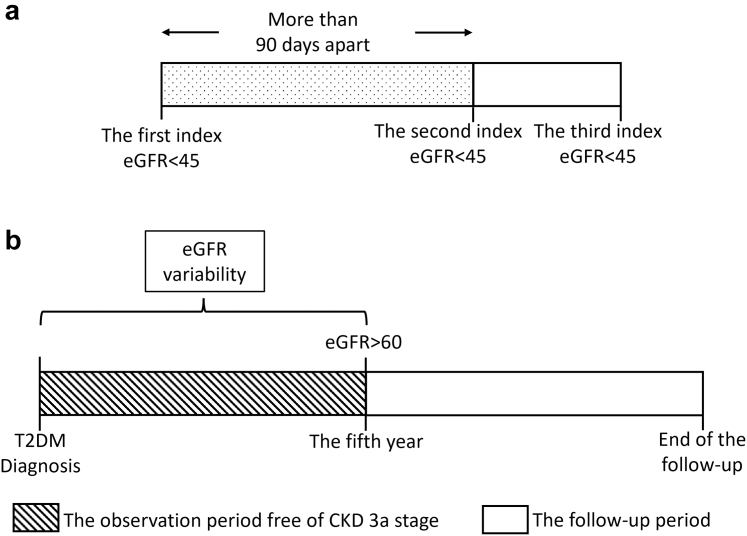
Study design. (a) Illustration of CKD stage G3b definition. The first eGFR index was defined as the onset of CKD stage G3b if all intervening values between this first index and the second remained below (45 + 2 × SD). Otherwise, the search proceeds to the interval between the second and third indices. (b) Illustration of the follow-up period. Follow-up began 5 years after diabetes diagnosis and continued until the occurrence of an event or censoring. The 5 years following diagnosis were used to detect eGFR variability and to confirm the absence of progression to stage 3a CKD. CKD, chronic kidney disease; eGFR, estimated glomerular filtration rate.

The CKD- Epidemiology Collaboration formula was used to estimate eGFR from SCr.^4^ CKD diagnosis date was defined following the CKD-Kidney Disease: Improving Global Outcomes guideline as eGFR < 60 ml/min per 1.73 m^2^ present on at least 2 occasions at least 90 days apart.^4^ CKD G3b stage date was also defined following the CKD-Kidney Disease: Improving Global Outcomes guideline as eGFR < 45 ml/min per 1.73 m^2^ present on at least 2 occasions at least 90 days apart. AKI episodes were identified using a previously validated algorithm based on relative increases in SCr.^7^ AKI was not an exclusion criterion at the individual level. Individuals with a history of AKI before the 5-year landmark were retained within the cohort, and baseline AKI history was included as an adjustment variable in all multivariable models. In contrast, SCr measurements occurring during AKI episodes were excluded at the test level from specific analytic steps, including determination of baseline CKD status, estimation of eGFR variability, and ascertainment of progression to CKD stage G3b. This approach was chosen to minimize inflation of eGFR variability driven by acute, transient changes in kidney function, while preserving the prognostic relevance of AKI history at the individual level. To avoid misclassification between AKI and CKD, eGFR values contained within AKI episodes were first removed from the longitudinal data. Because of infrequent or irregular measurements of SCr in routine healthcare data, the implementation of CKD-Kidney Disease: Improving Global Outcomes definition to determine CKD diagnostic dates and progression to further CKD G3b stage was tailored by allowing eGFR to fluctuate within a given band from the eGFR threshold (60 or 45 ml/min per 1.73 m^2^ respectively). Specifically, the size of the band was determined by fitting a regression spline model to the individual eGFR longitudinal data and calculating the SD of the residuals of the regression spline model. Based on the fact that 95% of eGFR values would deviate from the regression spline within 2SD of the residuals, the dates of CKD development and progression were determined by 2 occasions at least 90 days apart when eGFR was below 60, or 45 ml/min per 1.73 m^2^ (for progression to G3a or G3b stages respectively), and all the eGFR values between these 2 occasion were below 2SD from the specified threshold (below 60 + 2SD, or 45 + 2SD ml/min per 1.73 m^2^ respectively). An illustration of the CKD G3b onset date is presented in Figure 1a.

### Primary Outcome

Participants’ stage of CKD at recruitment was used to define the cohort for the analysis of the primary outcome. The primary analysis included people with preserved kidney function at diagnosis, defined as eGFR > 60 ml/min per 1.73 m^2^ at diagnosis and without 2 eGFR values below 60 ml/min per 1.73 m^2^, on 2 occasions more than 90 apart before diabetes diagnosis (this will be referred to as no-CKD at diagnosis throughout the manuscript). In addition, people who died or developed CKD G3a stage within the first 5 years from diabetes diagnosis were excluded from the study. The study, therefore, focused on individuals with preserved kidney function at diagnosis without rapid kidney decline within 5 years from diagnosis. An illustration of cohort selection is presented in Figure 1b. The primary outcome was defined as progression to G3b stage (defined as above, by the presence of 2 eGFR measures < 45 ml/min per 1.73 m^2^ on 2 occasions more than 90 days apart, with all values measured between these 2 instances below 45 + 2SD ml/min per 1.73 m^2^). The beginning of follow-up was defined as 5 years from date of diabetes diagnosis, and the event of interest was defined as progression to CKD G3b stage. The baseline of the study was defined as 5 years from diabetes diagnosis, continued until progression to CKD stage G3b, death, initiation of kidney replacement therapy, or extract date (December 31, 2021), whichever occurred first.

### Secondary Outcome

The secondary outcome of the study was the time to all-cause mortality, with follow-up time defined as the time from 5 years from diabetes diagnosis to date of death, or extract date (December 31, 2021), whichever occurred first.

### Definition of eGFR Variability

The eGFR variability was estimated for each individual by fitting a linear regression model to all available eGFR measurements obtained during the first 5 years following diabetes diagnosis. The SD of the model residuals (SDRes) was calculated once per individual and treated as a fixed exposure in subsequent analyses.^28^ As a result, the SDRes is a measure of eGFR variability independent of the eGFR decline captured by the slope. This measure generated continuous data which were then used to stratify the cohort into 4 groups (low, moderate, high, and very high variation) based on the quartiles of the continuous measures. This approach resulted in 4 balanced groups which stratified the population into first, second, third, and fourth quartiles groups (Q1, Q2, Q3, Q4) in terms of eGFR variability.

As sensitivity analyses, 2 measures of variation which have been used previously in clinical studies to measure variability in clinical and biochemical parameters, namely the coefficient of variation (CV) and coefficient of variation-variability independent of the mean (CV-VIM) were performed.^15^^,^^29^^,^^30^ The full definition of these 2 measures are provided in the Supplementary methods.

### Statistical Methods

Counts and proportions for categorical variables and means and SD for quantitative variables were used to describe patients’ characteristics at diabetes diagnosis or at 5 years from diagnosis and to allow comparison of these characteristics of people in the 4 groups determined based on the SDRes measure of eGFR variation.

The analysis of the primary outcome to evaluate the association between eGFR variation and time to progression to CKD stage G3b was conducted using cause-specific Cox proportional hazard model with death as a competing risk to the primary event defined as progression to CKD stage G3b. This approach was chosen to evaluate associations with cause-specific hazards rather than to estimate absolute risks. Association between eGFR variability and kidney function decline to CKD G3b stage was estimated in terms of HRs, first adjusted only for sex and age at diabetes diagnosis and then fully adjusted for all the other characteristics related to baseline kidney functions, such as eGFR at 5 years from diabetes diagnosis, eGFR slope estimated based on eGFR values in the 5 years from diabetes diagnosis, AKI history before 5 years from diagnosis (presence/absence of AKI), and number of SCr measures (calculated as number of the different days when a measure of SCr was taken) during the 5 years from diagnosis. A second group of variables included in the model consisted of physiological measures at 5 years from diagnosis, such as HbA1c, pulse pressure defined as the difference between systolic and diastolic pressure, as well as the BMI. For the primary CKD progression outcome, pulse pressure was selected as the blood pressure parameter as it reflects arterial stiffness and demonstrated stronger associations than systolic or diastolic blood pressure in exploratory models. Previous evidence has indicated that angiotensin converting enzyme inhibitors, angiotensin receptor blockers, and sodium-glucose cotransporter 2 inhibitors can induce variation in GFR through their mechanism of action.^31^ As a result, a binary variable indicated whether individual people were or have been in receipt of these medications in the period up to 5 years from diagnosis, was created and included in the model. Finally, the model was adjusted for cardiovascular disease related comorbidities developed up to the 5 years from diagnosis including congestive heart failure, coronary artery disease, peripheral vascular disease, and cerebrovascular disease.

For analysis of time to all-cause mortality a similar analysis approach was implemented with the exception that the mean arterial pressure, which appeared highly significant, was used in the fully adjusted model instead of the pulse pressure.

### Selection Bias and Testing Frequency

As SCr testing was performed as part of routine clinical care rather than a protocolized schedule, testing frequency may reflect underlying morbidity, healthcare utilization, or clinician concern. To address potential selection bias arising from differential surveillance intensity, the number of distinct SCr measurement days during the 5-year exposure window was quantified for each individual and included as an adjustment variable in all fully adjusted models. This approach aimed to account, in part, for differences in healthcare contact and clinical complexity that may influence both eGFR variability and subsequent kidney outcomes.

### Missing Data

Routine measures in the 6 months before the 5 years from diabetes diagnosis such as HbA1c, blood pressure, and BMI were missing in proportion varying from 7.6% for HbA1c to 9.1% for blood pressure, and 13.6% for BMI. Multiple imputations were used to impute the missing values using the predictive mean matching method implemented as part of the *mice* package in R (R Foundation, Vienna, Austria). This was done based on 5 imputations assuming data were missing at random. Main analysis was performed on the imputed dataset, which was based on a pooled analysis of 5 imputed datasets.^32^ Completes cases analysis was performed as sensitivity analysis for comparison.

Data linkage and analysis was carried out using R 4.1 statistical software on the SDRN Safe Haven platform.

## Results

### Baseline Characteristics

The estimated population of Scotland is approximately 5.45 million people of which around 6.5% have diabetes.^33^ There were 34,840,264 SCr tests from 529,936 individuals with type 1 or T2DM between January 1985 to December 2021. Of these, 1,121,174 (3.2%) were flagged as AKI cases which were further grouped into 3,053,175 AKI episodes. The number of SCr tests within the AKI episodes further increased at 1,963,945 (5.64%) measures, which were removed from the dataset before evaluation of date of development of CKD and progression to further CKD stages.

**Figure 2:**
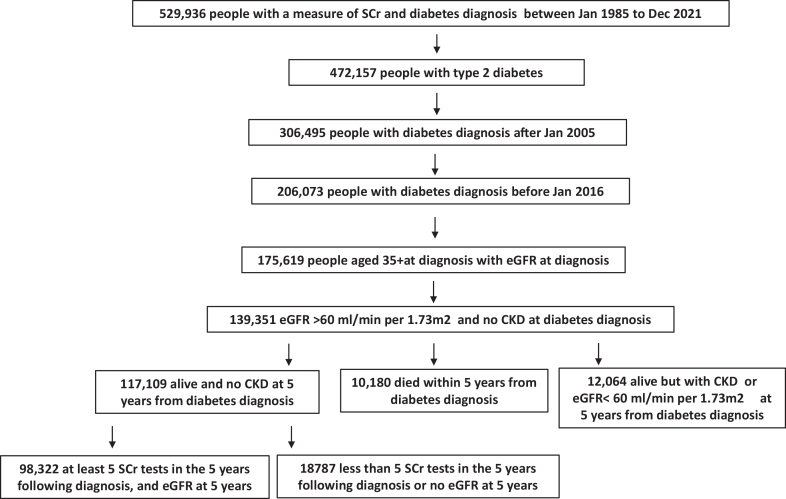
Flowchart of cohort selection.

The flowchart in Figure 2 illustrates the steps followed to derive the cohort. Of the total of 529,936 individuals in the SDRN database, 472,157 people had T2DM. There were 306,495 people with a diagnosis of T2DM after January 2005, of which 206,073 had a diagnosis before January 2016, and at least 6 years of follow-up. Of these, 175,619 individuals were aged ≥ 35 at diagnosis and had a SCr measure, and implicitly an estimated GFR at diagnosis, of which 139,351 had an eGFR > 60 ml/min per 1.73 m^2^ and did not have CKD at diagnosis (defined as no 2 eGFR values more than 90 apart below 60 ml/min per 1.73 m^2^ before diabetes diagnosis). Of these, 117,109 were alive at 5 years from diabetes diagnosis, and had not developed CKD at 5 years from diabetes diagnosis. Among these individuals, 98,322 had at least 5 SCr tests (on 5 different occasions/days) in the 5 years follow-up from diabetes diagnosis and had a measure of SCr in the year preceding the 5 years from diagnosis. These individuals formed the study cohort.

Table 1 shows the summary statistics of baseline characteristics of the cohort at diabetes diagnosis or at 5 years from diabetes diagnosis and how these statistics vary for the different eGFR SDRes variability-based quartile groups. Of the 98,322 people in the study, 59,491 (60.5%) were men, and the distribution varied slightly in the eGFR SDRes groups, with fewer men experiencing high and very high SDRes as compared with women. Mean age was 57.07 (SD: 10.28) years, with a lower mean age in the fourth eGFR SDRes variability-based quartile group compared with the other 3 groups. Mean eGFR at 5 years was 88.58 (SD: 12.51) ml/min per 1.73 m^2^, with a higher mean eGFR in the fourth eGFR SDRes variability-based quartile group as compared with the other 3 groups. 4219 (4.29%) individuals experienced an AKI episode before 5-year follow-up from diagnosis, with the proportion of people experiencing AKI increasing being higher in the fourth eGFR SDRes variability-based quartile group as compared with the other 3 groups. On average, people had 12.78 (SD: 8.80) distinct SCr tests during the 5-year post diabetes diagnosis, and this number increased with the increase in eGFR variability. Pulse pressure at 5 years slightly decreased with the increase of eGFR variability, whereas the mean BMI and HbA1C slightly increased with the increased of eGFR variability. Similarly, the proportion of people in receipt of angiotensin converting enzyme inhibitors/angiotensin receptor blockers, and sodium-glucose cotransporter 2 inhibitor medication before the 5 years from diabetes baseline increased with the increase of eGFR SDRes based variability, and a similar trend was observed in the proportion of people with CVD related comorbidities.

**Table 1:** Summary statistics of the cohort characteristics at type 2 diabetes diagnosis or at 5 yrs from diagnosis

Variables	Q1 group, least variable (*n* = 24,648)	Q2 group (*n* = 24,591)	Q3 group (*n* = 24,572)	Q4 group, most variable (*n* = 24,511)	Overall (*N* = 98,322)
Gender, male, *n* (%)	15856 (64.3%)	15314 (62.2%)	14802 (60.2%)	13519 (55.2%)	59491 (60.5%)
Age at diagnosis (yrs) mean (SD)	57.61 (10.42)	57.85 (10.45)	57.48 (10.12)	55.33 (9.92)	57.07 (10.28)
eGFR at 5 yrs from diagnosis	93.41 (11.66)	88.52 (12.76)	86.30 (12.06)	86.00 (12.12)	88.58 (12.51)
eGFR slope from diagnosis to 5 yrs	−0.31 (1.71)	−0.04 (2.27)	0.21 (2.71)	0.29 (3.17)	0.04 (2.53)
AKI status at 5 yrs (AKI vs. non-AKI)	562 (2.28%)	727 (2.96%)	966 (3.93%)	1964 (8.01%)	4219 (4.29%)
No. of SCr measures 5 yrs	10.49 (5.98)	12.23 (7.45)	13.61 (9.20)	14.87 (11.09)	12.78 (8.80)
Pulse pressure at 5 yrs	56.72 (13.14)	56.61 (13.43)	56.12 (13.30)	55.39 (13.37)	56.22 (13.32)
BMI at 5 yrs from diagnosis, mean (SD)	31.97 (6.46)	32.10 (6.44)	32.23 (6.42)	32.90 (6.81)	32.30 (6.55)
HbA1c at 5 yrs from diagnosis, mean (SD)	57.35 (15.64)	57.42 (16.01)	57.85 (16.45)	59.02 (17.85)	57.91 (16.52)
ACE/ARB use at 5 yrs from diagnosis	14569 (59.10%)	15239 (61.97%)	15735 (64.04%)	15878 (64.78%)	61421 (62.47%)
SGLT2 use at 5 yrs from diagnosis	1258 (5.10%)	1273 (5.18%)	1328 (5.40%)	1517 (6.20%)	5376 (5.47%)
CHF at 5 yrs from diagnosis	465 (1.89%)	628 (2.52%)	758 (3.05%)	963 (3.93%)	2798 (2.85%)
PVD at 5 yrs from diagnosis	370 (1.50%)	404 (1.64%)	416 (1.69%)	492 (2.01%)	1682 (1.71%)
CAD at 5 yrs from diagnosis	4430 (17.97%)	4983 (19.94%)	5406 (22%)	5598 (22.84%)	20337 (20.68%)
CD at 5 yrs from diagnosis	5823 (23.62%)	6416 (26.09%)	6992 (28.46%)	7391 (30.15%)	26622 (27.08%)
Outcome event					
CKD stage G3b, *N* (%)	441 (1.79%)	746 (3.03%)	882 (3.59%)	1027 (4.19%)	3096 (3.15%)
Follow-up time mean (SD) in yrs	5.45 (2.06)	5.71 (3.02)	5.85 (3.10)	5.86 (3.16)	5.72 (3.04)
Death with noCKD stage G3b, *N* (%)	2944 (11.94%)	3283 (13.35%)	3374 (13.73%)	3300 (13.71%)	12981 (13.20%)
All death *N* (%)	3088 (12.52%)	3536 (14.38%)	3682 (14.98%)	3678 (15.01%)	13984 (14.22%)

AKI, acute kidney injury; ACE/ARB, angiotensin converting enzyme/angiotensin receptor blockers; BMI, body mass index; CAD, coronary artery disease; CD, cerebrovascular disease; CHF, congestive heart failure; eGFR, estimated glomerular filtration rate; PVD, peripheral vascular disease.

The baseline characteristics of people excluded from the study either because they died within 5 years from diagnosis, developed CKD, or had less than 5 SCr measures in the 5 years follow-up are presented in the Supplementary Table S1. Most notably, the results show that 36.72% of people who developed CKD stage G3a (eGFR < 60 ml/min per 1.73 m^2^) within 5 years from diagnosis and who were excluded from the study, progressed to stage CKD G3b during the follow-up time.

### Primary Analysis of Time to Progression to CKD G3b Stage

Mean follow-up time was 5.72 years (SD = 3.04), in addition to the 5-year initial follow-up period from diabetes diagnosis. In total, there were 3096 (3.15%) people who developed CKD stage G3b until end of follow-up time. There was an increase in the number of people who developed CKD stage G3b with a higher eGFR SDRes based variability from 441 (1.79%) in the first quartile (Q1, lowest variability) group to 1027 (4.19%) in the fourth quartile (highest variability) eGFR variability group. Overall, 13,984 (14.22%) people died by the end of the follow-up time, with some variation in mortality rate among the 4 eGFR variability quartile groups.

As shown in Table 2, the results of the sex and age adjusted model and fully adjusted model indicated that people in the second, third, and fourth quartile (Q2, Q3, Q4) eGFR variability groups were at a significant increased risk of developing CKD G3b stage as compared with people in the first quartile (Q1) group. The hazard ratio gradually increased from the Q2 versus Q1 to the Q4 versus Q1 groups in the sex and age adjusted analysis, as well as the fully adjusted analysis. Specifically, the sex and age adjusted analysis indicated that people with moderate eGFR variability (Q2) are HR: 1.56 (95% CI: 1.38–1.75) more at risk of developing CKD G3b than people in the low group (Q1), the figure for the high (Q3 vs. Q1) and very high (Q4 vs. Q1) eGFR variability group increasing to HR: 1.85 (95% CI: 1.65–2.08) and HR: 2.56 (95% CI: 2.29–2.86) for the very high versus low group (Q4 vs. Q1) comparison. The association decreased but remained significantly high after adjusting the model for all the baseline variables in the study (HR: 1.15 [95% CI: 1.03–1.30] for the moderate versus low comparison (Q2 vs. Q1) , HR: 1.27 (95% CI: 1.13–1.43) for the high versus low group comparison (Q3 vs. Q1), and HR: 1.57 (95% CI: 1.40–1.77) for the very high versus low group (Q4 vs. Q1) comparison). Additionally, all the variables included in the model were significantly associated with the development of CKD G3B stage (Supplementary Table S2).

**Table 2:** Quartile of eGFR variability and risks of outcomes

Quartile of SDRes	Age and sex adjusted model HR (95% CI)	Age and sex, eGFR slope, eGFR at 5 yrs, AKI adjusted model HR (95% CI)	Fully adjusted model HR (95% CI)
Stage 3b CKD			
Q1	Reference	Reference	Reference
Q2	1.56 (1.38–1.75)^a^	1.19 (1.06–1.35)^b^	1.16 (1.03–1.30)^c^
Q3	1.85 (1.65–2.08)^a^	1.33 (1.19–1.50)^a^	1.27 (1.13–1.43)^a^
Q4	2.56 (2.29–2.86)^a^	1.69 (1.50–1.90)^a^	1.57 (1.40–1.77)^a^
All-cause mortality			
Q1	Reference	Reference	Reference
Q2	1.05 (1.005–1.106)^c^	1.14 (1.08–1.20)^a^	1.12 (1.07–1.18)^a^
Q3	1.12 (1.064–1.171)^a^	1.23 (1.17–1.29)^a^	1.18 (1.13–1.25)^a^
Q4	1.35 (1.288–1.419)^a^	1.47 (1.40–1.155)^a^	1.40 (1.33–1.48)^a^

ACEi/ARB, angiotensin-converting enzyme inhibitors/ angiotensin receptor blockers; AKI, acute kidney injury; BMI, body mass index; CI, confidence interval; CKD, chronic kidney disease; eGFR, estimated glomerular filtration rate; HbA1c, hemoglobin A1c; HR, hazard ratio; SCr, serum creatinine; SDRes, SD of the model residuals.

Results of the Cox survival model for the analysis of the primary and secondary outcomes (progression to stage 3b CKD and all-cause mortality) Results are reported as hazard ratios compared with the reference group adjusted for age and sex; age, sex, eGFR slope, eGFR at 5 years and AKI and fully adjusted for eGFR at baseline, eGFR slope, AKI history, the number of SCr measures, HbA1c, pulse pressure, BMI, ACEi/ARB use, congestive heart failure, coronary artery disease, peripheral vascular disease and cerebrovascular disease.

a *P*-value < .001

b .001 < *P*-value < .01

c .01 < *P*-value < .05

The analysis of the secondary outcome showed significant associations between eGFR variability and all-cause mortality for both sex and age adjusted model and the fully adjusted model (Table 2). The hazard ratio for all-cause mortality increased gradually from Q2 versus Q1 to Q4 versus Q1 groups in both models. Specifically, in the fully adjusted model people with moderate eGFR variability (Q2) were HR: 1.12 (95% CI: 1.07–1.18) more at risk of developing CKD G3b than people in the low group (Q1), the figure for the high (Q3 vs. Q1) and very high (Q4 vs. Q1) eGFR variability group increasing to HR: 1.18 (95% CI: 1.13–1.25) and HR: 1.40 (95% CI: 1.33–1.48). The full results of the secondary analysis are presented in the Supplementary Table S3.

### Sensitivity Analysis

The complete cases analysis was performed as sensitivity analysis to evaluate the robustness of results after multiple imputations and is presented in the Supplementary Table S2. Without exception the sensitivity analysis showed very consistent results between the imputed data analysis and the complete cases analysis with very little difference in HRs for the association of eGFR variability across all 3 definitions.

Also, sensitivity analysis of eGFR variability based on the CV and coefficient of variation, with variability independent of the mean (CV-VIM) showed similar associations with time to development of G3b stage CKD (Supplementary Table S4). For instance, evaluation of eGFR CV based variability adjusted for age and gender indicated that people with moderate eGFR variability (Q2) present a HR: 1.62 (95% CI: 1.41–1.85) of developing CKD G3b than people in the low group (Q1), the figure for the high eGFR variability group (Q3) increasing to HR: 2.32 (95% CI: 2.05–2.64) and HR: 3.13 (95% CI: 2.77–3.55) for the very high (Q4) versus low group (Q1) comparison. The association decreased and was no longer significant for the Q2 versus Q1 group comparison, after adjusting the model for all the baseline variables in the study (HR: 1.08, 95% CI: 0.93–1.24), but they remained significant for the Q3 versus Q1 group comparison, (HR: 1.30, 95% CI: 1.13–1.49), and the Q4 versus Q1 group comparison respectively, (HR: 1.59, 95% CI: 1.39–1.83).

## Discussion

In this nationwide observational study, we have demonstrated that eGFR variability in the first 5 years after the diagnosis of T2DM is associated with kidney disease progression in people with preserved kidney function. Sensitivity analysis was conducted by estimating eGFR variability with different measures confirming the robustness of our findings. This association persists after adjustment for multiple variables and those in the highest quartile of eGFR variability had more than 1 and a half times the risk compared with those in the lowest quartile.

Higher eGFR variability was more frequently observed among individuals with greater testing intensity, previous AKI, and cardiovascular comorbidity, supporting the interpretation that variability reflects underlying clinical complexity, hemodynamic instability, and healthcare utilization rather than kidney function alone. Several previous studies have shown that eGFR variability predicts mortality and CKD progression in individuals with impaired kidney function.^20^, ^21^, ^22^, ^23^ Al-Aly *et al.*^21^ examined a cohort of 51,304 US veterans with eGFR less than 60 ml/min per 1.73 m^2^. They showed that greater variability in eGFR was independently associated with increased mortality. In a cohort of 70,598 patients from the Veterans Health Administration with diabetes and CKD Stage 3 and 4, eGFR variability was associated with elevated risk of dialysis and death even after controlling for slopes in CKD progression and other clinical factors.^22^ Recently, in an analysis of the chronic renal insufficiency cohort with an eGFR between 20 and 70 ml/min per 1.73 m^2^, time updated eGFR variability was associated with a higher risk of cardiovascular disease, end-stage kidney disease, and all-cause mortality.^20^ Our study extends this knowledge further by demonstrating that eGFR variability is associated with CKD progression even in individuals with diabetes and preserved kidney function. This highlights its potential for early identification of high-risk individuals within the diabetic population, including those who do not experience progressive eGFR decline but may still face an increased risk of CKD progression. There are several different mechanisms which may contribute to the observed associations. Firstly, impaired autoregulation because of diabetes can render the kidneys more vulnerable to hemodynamic stress. Secondly, eGFR variability may result from undiagnosed or subclinical episodes of AKI, which are known to accelerate CKD progression. In this study, we have excluded AKI which meets the biochemical definition. However, smaller fluctuations in eGFR may reflect impaired renal autoregulation and routine exposure to factors that compromise renal homeostasis. These factors encompass both renal issues, such as altered renal hemodynamics and nephron loss, and extrinsic influences, including changes in volume status or comorbidities. Although the mechanisms behind eGFR variability may resemble those of blood pressure variability, a study suggests that the 2 act as independent predictors of risk changes in patients.^25^ In this study, the independent association of eGFR variability with CKD progression indicates that it may represent a variety of unfavorable factors and exposures that are not easily captured by traditional clinical definitions and measurements. Our study extends these findings by examining eGFR variability in a nationwide, unselected, real-world population, specifically restricted to individuals with preserved kidney function at a 5-year landmark, thereby isolating variability independent of early CKD or rapid decline. Currently, there is an emerging shift in the perspective on diabetic kidney disease, with a growing emphasis on variable risk factors rather than on albuminuria and glucose-centered assessments. A multidimensional risk assessment of CKD progression is essential, especially in the diabetes population.^34^

This study has several strengths. Firstly, the cohort was formed using the national diabetes registry which includes over 99% of individuals with diabetes in Scotland and provides access to multiple high quality routine healthcare databases. Secondly, AKI episodes were removed to ensure that we captured true variability rather than AKI episodes which are known to be associated with future development of CKD. Thirdly, a robust algorithm was developed and applied to the longitudinal eGFR data to identify the date of CKD development and progression to further CKD stages. Finally, we employed 3 different methods of assessing eGFR variability which yielded similar results ensuring robustness of our findings. There are 2 constitutive elements when evaluating the variability—the variation in the longitudinal trend and the variation in the fluctuations below and above the trend. Although it is well established that decline in eGFR measured by the eGFR slope is significantly associated with development and progression of CKD,^35^ there is little evidence showing that the fluctuations below and above the trend or slope are also associated with an increased risk of CKD development and progression. Both CV and coefficient of variation-variability independent of the mean measures are affected by the variation in eGFR trend, and therefore, it is difficult to separate the effect of the actual fluctuations from the effect because of eGFR decline measured by the slope. We therefore implemented an alternative method as our primary outcome to evaluate eGFR variability independent of the slope.

An important limitation of this study is the potential for selection bias and residual confounding inherent to longitudinal electronic health record data. Individuals undergoing more frequent SCr testing are likely to differ systematically from those tested less often, reflecting greater illness burden, intercurrent events, or clinician-driven surveillance. Although we adjusted for testing frequency, AKI history, cardiovascular comorbidities, and other clinical variables, residual confounding related to unmeasured illness severity, healthcare-seeking behavior, and inpatient versus outpatient sampling cannot be fully excluded. This limitation is particularly relevant when the exposure of interest is derived from repeated measurements. Furthermore, albuminuria was not included in the model as there were insufficient measures during our period of interest. Albuminuria is a well-established predictor of CKD progression and may modify or mediate the association between eGFR variability and outcomes. The absence of albuminuria data therefore represents an important limitation, and future studies should evaluate whether eGFR variability provides prognostic information beyond albuminuria-based risk stratification. In addition, BMI was assessed only at the 5-year landmark baseline, and unmeasured changes in muscle mass during follow-up may have influenced creatinine-based eGFR variability. Moreover, inclusion of both inpatient and outpatient creatinine measurements may have influenced estimates of eGFR variability, as inpatient values are more likely to reflect acute illness. Finally, while our cohort is likely to be representative of the UK population with diabetes, our findings may not be generalizable to other ethnicities or countries without universal healthcare.

Our findings have important clinical implications. From a clinical perspective, eGFR variability may serve as a readily available prognostic marker to help identify individuals with T2DM who are at higher risk of kidney disease progression, even when average eGFR remains within the normal range. These findings should be interpreted as supporting risk stratification rather than a causal therapeutic target.

Notably, variability in eGFR over time can provide prognostic information beyond a single eGFR measurement, particularly in individuals with preserved kidney function and absent or unmeasured albuminuria. Incorporating eGFR variability into routine assessment may therefore aid in identifying individuals who warrant closer monitoring, stricter optimization of blood pressure and glycemic control, and consideration of earlier initiation or intensification of established reno-protective therapies, such as angiotensin-converting enzyme inhibitors, angiotensin receptor blockers, or sodium–glucose co-transporter-2 inhibitors, in accordance with clinical guidelines.

### Conclusion

In conclusion, this large-scale, population-based study demonstrates that non-AKI eGFR variability is a predictor for CKD progression even among individuals with preserved kidney function at baseline. These findings underscore the prognostic value of eGFR variability as an early marker of renal risk and highlight the potential for incorporating these metrics into routine clinical risk stratification. Future research should explore the underlying mechanisms driving eGFR fluctuations and evaluate whether interventions targeting variability can mitigate CKD progression in this high-risk population.

## Disclosure

SB reports research funding from AstraZeneca and consultancy fees from AstraZeneca, GSK, Bayer, and Stada UK. HMC reports research funding from Sanofi, consulting fees from Bayer, Roche, Novo Nordisk and Sanofi, travel support from Novo Nordisk and stock ownership in Roche and Bayer. SH, QY, SL, LAKB, SJM, and ERP report no competing interests.

## Supplementary Material

Supplementary File (PDF) **Supplementary Methods. Table S1.** Descriptive statistics of people excluded from the analysis because of death or CKD development within 5 years from diabetes diagnosis, or less than 5 SCr tests in the 5 years from diagnosis. **Table S2.** Full results of the cause specific Cox survival model for time to G3b stage CKD, showing hazard ratio and 95% confidence interval adjusted for age and gender, complete case analysis of the fully adjusted model and fully adjusted model after multiple imputation, with eGFR variation derived following the SDRes based definition. Sensitivity analysis comparing complete case analysis result to multiple imputation results of Cox survival model for time to progression to G3b stage, with eGFR variation derived following the SDRes based definition. **Table S3.** Secondary analysis: results of the Cox survival model for time to all-cause mortality, showing hazard ratios and 95% confidence intervals adjusted for age and sex, fully adjusted model after multiple imputation the fully adjusted model for complete case analysis, with eGFR variation derived following the SDRes based definition. **Table S4.** Quartile of eGFR variability and risks of outcomes. STROBE checklist.

## Footnotes

Supplementary File (PDF)

**Supplementary Methods.**
**Table S1.** Descriptive statistics of people excluded from the analysis because of death or CKD development within 5 years from diabetes diagnosis, or less than 5 SCr tests in the 5 years from diagnosis. **Table S2.** Full results of the cause specific Cox survival model for time to G3b stage CKD, showing hazard ratio and 95% confidence interval adjusted for age and gender, complete case analysis of the fully adjusted model and fully adjusted model after multiple imputation, with eGFR variation derived following the SDRes based definition. Sensitivity analysis comparing complete case analysis result to multiple imputation results of Cox survival model for time to progression to G3b stage, with eGFR variation derived following the SDRes based definition. **Table S3.** Secondary analysis: results of the Cox survival model for time to all-cause mortality, showing hazard ratios and 95% confidence intervals adjusted for age and sex, fully adjusted model after multiple imputation the fully adjusted model for complete case analysis, with eGFR variation derived following the SDRes based definition. **Table S4.** Quartile of eGFR variability and risks of outcomes. STROBE checklist.
