# Supplementary material for: The Impact of Non-AKI eGFR Variability on CKD Progression in Individuals With Type 2 Diabetes and Preserved Kidney Function
Source: Kidney Int Rep. 2026 Jun 18;11(9):106667. doi: 10.1016/j.ekir.2026.106667 (PMC13375920; doi:10.1016/j.ekir.2026.106667)
Supplement: Supplementary File (PDF) — Supplementary Methods. Table S1. Descriptive statistics of people excluded from the analysis because of death or CKD development within 5 years from diabetes diagnosis, or less than 5 SCr tests in the 5 years from diagnosis. Table S2. Full results of the cause specific Cox survival model for time to G3b stage CKD, showing hazard ratio and 95% confidence interval adjusted for age and gender, complete case analysis of the fully adjusted model and fully adjusted model after multiple imputation, with eGFR variation derived following the SDRes based definition. Sensitivity analysis comparing complete case analysis result to multiple imputation results of Cox survival model for time to progression to G3b stage, with eGFR variation derived following the SDRes based definition. Table S3. Secondary analysis: results of the Cox survival model for time to all-cause mortality, showing hazard ratios and 95% confidence intervals adjusted for age and sex, fully adjusted model after multiple imputation the fully adjusted model for complete case analysis, with eGFR variation derived following the SDRes based definition. Table S4. Quartile of eGFR variability and risks of outcomes. STROBE checklist. [file mmc1.pdf]

Supplementary Materials

Supplementary Methods

Definition of eGFR variation based on the coefficient of variation (CV) and coefficient of variation independent of the mean (CV-VIM)

The ***Coefficient of Variation (CV)*** was calculated using the standard definition of the ratio between the eGFR standard deviation (SD) and eGFR mean of all measures in the five-year interval.

$$CV = \frac{SD}{mean}$$

A second measure of variation known as **Coefficient of Variation -Variability Independent of the Mean (CV-VIM)** was defined as:

$$CV - VIM = \frac{SD}{mean^{\alpha}} \times 100$$

Where the coefficient α is the slope estimate calculated by regressing the individual $\log(SD)$ versus $\log(mean)$ of eGFR values in the population.

Supplementary Table S1: Descriptive statistics of people excluded from the analysis due to death or CKD development within 5 years from diabetes diagnosis, or less than five SCr tests in the five years from diagnosis.

Variables	Died within 5 years from diabetes diagnosis (N=10180)	Alive and progressed to CKD within 5 years from diabetes diagnosis (N=12064)	Less that 5 SCr tests in the 5 years from diabetes diagnosis (N=9038)
Sex Male, N (%)	6417 (63.04%)	6285 (52.14%)	11678 (62.16%)
Age at diagnosis (years)	67.91 (10.82)	66.32 (8.86)	54.86 (10.61)
Outcome event: CKD stage G3b, N (%)	279 (2.75%)	4426 (36.72%)	458 (2.44%)

Supplementary Table S2: Full results of the cause specific Cox survival model for time to G3b stage CKD, showing Hazard ratio and 95% Confidence Interval adjusted for age and gender, complete case analysis of the fully adjusted model and fully adjusted model after multiple imputation, with eGFR variation derived following the SDRes based definition. Sensitivity analysis comparing complete case analysis result to multiple imputation results of Cox survival model for time to progression to G3b stage, with eGFR variation derived following the SDRes based definition

Model variables	HR (95% CI) age/gender adjusted model (N= 98,322)	HR (95% CI) adjusted model after multiple imputations (N= 98,322)	Sensitivity analysis HR (95% CI) adjusted model, complete case analysis (N= 87860)
eGFR SDRes variability (Q2 vs Q1)	1.557 (1.384-1.752)***	1.155 (1.025-1.303)*	1.141 (1.007-1.292)*
eGFR SDRes variability (Q3 vs Q1)	1.853 (1.653-2.078)***	1.273 (1.131-1.433)***	1.261 (1.116-1.425)***
eGFR SDRes variability (Q4 vs Q1)	2.559 (2.287-2.864)***	1.572 (1.397-1.769)***	1.562 (1.382-1.766)***
Gender (Female vs Male)	0.974 (0.906-1.047)	0.926 (0.86-0.998)*	0.916 (0.849-0.989)*
Age at diagnosis (years)	1.076 (1.072-1.08)***	1.049 (1.044-1.054)***	1.049 (1.044-1.054)***
eGFR at 5 yrs per 1 unit increase	0.942 (0.938-0.946)***	0.946 (0.943-0.95)***	0.946 (0.942-0.95)***
eGFR slope per 1 unit increase	0.974 (0.959-0.989)***	0.973 (0.958-0.989)***	0.975 (0.96-0.991)**
AKI history at 5 yrs (AKI vs non-AKI)	1.634 (1.407-1.899)***	1.204 (1.024-1.416)*	1.232 (1.045-1.453)*
No of SCr tests from diagnosis to 5 yrs	1.016 (1.013-1.019)***	1.009 (1.006-1.012)***	1.009 (1.005-1.013)***
Pulse pressure at 5 yrs per 1 unit increase	1.009 (1.006-1.011)***	1.009 (1.006-1.012)***	1.009 (1.006-1.011)***
BMI at 5 yrs per 1 unit increase	1.03 (1.024-1.036)***	1.022 (1.015-1.028)***	1.022 (1.016-1.028)***
HbA1c at 5 yrs per 1 unit increase	1.011 (1.008-1.013)***	1.011 (1.008-1.013)***	1.01 (1.008-1.013)***
ACE/ARBs status use at 5 yrs (ever vs never)	1.762 (1.621-1.916)***	1.450 (1.331-1.580)***	1.441 (1.318-1.576)***
SGLT2 status use at 5 yrs (ever vs never)	0.43 (0.262-0.704)***	0.368 (0.225-0.604)***	0.392 (0.239-0.642)***
Congestive Heart Failure at 5 yrs	2.079 (1.789-2.416)***	1.719 (1.468-2.013)***	1.705 (1.457-1.994)***

Peripheral Vascular Disease at 5 yrs	1.723 (1.398-2.123)***	1.689 (1.372-2.081)***	1.762 (1.421-2.185)***
Coronary Artery Disease at 5 yrs	1.246 (1.146-1.355)***	1.176 (1.08-1.282)***	1.193 (1.094-1.302)***
Cerebrovascular disease at 5 yrs	1.132 (1.047-1.224)***	1.085 (1.002-1.175)*	1.100 (1.014-1.194)*

Supplementary Table S3: Secondary analysis: results of the Cox survival model for time to all-cause mortality, showing Hazard Ratios and 95% Confidence Intervals adjusted for age and sex, fully adjusted model after multiple imputation the fully adjusted model for complete case analysis, with eGFR variation derived following the SDRes based definition.

Variables	HR (95% CI) age/gender adjusted model (N=84549)	HR (95% CI) adjusted model after multiple imputations (N=84549)	HR (95% CI) adjusted model, complete cases analysis (N=70330)
eGFR SDRes variability (Q2 vs Q1)	1.054 (1.005-1.106)*	1.121 (1.067-1.178)***	1.104 (1.046-1.164)***
eGFR SDRes variability (Q3 vs Q1)	1.117 (1.064-1.171)***	1.184 (1.126-1.246)***	1.169 (1.107-1.234)***
eGFR SDRes variability (Q4 vs Q1)	1.352 (1.288-1.419)***	1.401 (1.329-1.477)***	1.383 (1.307-1.464)***
Sex (Female vs Male)	0.824 (0.796-0.853)***	0.848 (0.819-0.879)***	0.819 (0.788-0.85)***
Age at diagnosis (years)	1.09 (1.088-1.092)***	1.103 (1.1-1.105)***	1.101 (1.098-1.104)***
eGFR at baseline per 1 unit increase	1.018 (1.016-1.02)***	1.023 (1.02-1.025)***	1.021 (1.018-1.023)***
eGFR slope per 1 unit increase	0.973 (0.966-0.98)***	0.967 (0.96-0.974)***	0.966 (0.958-0.974)***
AKI history at 5 yrs (AKI vs non-AKI)	1.791 (1.677-1.913)***	1.271 (1.185-1.363)***	1.212 (1.121-1.311)***
No of SCr tests from diagnosis to 5 yrs	1.018 (1.017-1.019)***	1.014 (1.013-1.015)***	1.013 (1.012-1.014)***
MAP at 5 yrs per 1 unit increase	0.991 (0.989-0.993)***	0.993 (0.991-0.995)***	0.994 (0.992-0.996)***
BMI at 5 yrs per 1 unit increase	1.004 (1.001-1.007)*	1.002 (0.998-1.005)‡	1.003 (0.999-1.006)‡
HbA1c at 5 yrs per 1 unit increase	1.004 (1.003-1.005)***	1.003 (1.002-1.004)***	1.003 (1.002-1.005)***
ACE/ARBs use at 5 yrs (ever vs never)	1.087 (1.049-1.126)***	1.017 (0.98-1.055)	1.038 (0.998-1.08)
SGLT2 use at 5 yrs (ever vs never)	1.014 (0.879-1.171)	0.893 (0.773-1.031)	0.934 (0.803-1.087)
CHF at 5 yrs from diagnosis	1.877 (1.752-2.01)***	1.702 (1.588-1.824)***	1.654 (1.533-1.785)***
PVD at 5 yrs from diagnosis	1.514 (1.373-1.67)***	1.447 (1.311-1.596)***	1.401 (1.257-1.561)***
CAD at 5 yrs from diagnosis	1.272 (1.224-1.322)***	1.235 (1.188-1.284)***	1.239 (1.188-1.292)***
CD at 5 yrs from diagnosis	1.369 (1.321-1.418)***	1.296 (1.25-1.343)***	1.287 (1.238-1.337)***

Supplementary Table S4 Quartile of eGFR variability and risks of outcomes Sensitivity analysis showing Hazard Ratios and 95% Confidence Intervals of the Cause Specific Cox survival model, adjusted for age and gender, and fully adjusted model after multiple imputation and complete case analysis, with eGFR variation derived following the CV and CV-VIM based definition respectively.

	**Age and sex adjusted model HR (95% CI)**	**Fully adjusted model HR (95% CI)**
**CV groups**		
Q1	Reference	Reference
Q2	1.617 (1.411-1.854)***	1.076 (0.934-1.24)
Q3	2.324 (2.045-2.642)***	1.296 (1.129-1.487)***
Q4	3.133 (2.768-3.547)***	1.592 (1.388-1.826)***
**CV-VIM groups**		
Q1	Reference	Reference
Q2	1.572 (1.404-1.76)***	1.158 (1.032-1.3)*
Q3	1.908 (1.697-2.146)***	1.308 (1.16-1.475)***
Q4	2.417 (2.16-2.704)***	1.585 (1.411-1.779)***

STROBE Statement—checklist of items that should be included in reports of observational studies

	Item No.	Recommendation	Page No.	Relevant text from manuscript
**Title and abstract**	1	(*a*) Indicate the study's design with a commonly used term in the title or the abstract	1	
		(*b*) Provide in the abstract an informative and balanced summary of what was done and what was found	5,6	
**Introduction**				
Background/rationale	2	Explain the scientific background and rationale for the investigation being reported	7	
Objectives	3	State specific objectives, including any prespecified hypotheses	8	
**Methods**				
Study design	4	Present key elements of study design early in the paper	8	
Setting	5	Describe the setting, locations, and relevant dates, including periods of recruitment, exposure, follow-up, and data collection	8	
Participants	6	(*a*) *Cohort study*—Give the eligibility criteria, and the sources and methods of selection of participants. Describe methods of follow-up<br>*Case-control study*—Give the eligibility criteria, and the sources and methods of case ascertainment and control selection. Give the rationale for the choice of cases and controls<br>*Cross-sectional study*—Give the eligibility criteria, and the sources and methods of selection of participants	8	
		(*b*) *Cohort study*—For matched studies, give matching criteria and number of exposed and unexposed<br>*Case-control study*—For matched studies, give matching criteria and the number of controls per case	n/a	
Variables	7	Clearly define all outcomes, exposures, predictors, potential confounders, and effect modifiers. Give diagnostic criteria, if applicable	10,11	
Data sources/ measurement	8*	For each variable of interest, give sources of data and details of methods of assessment (measurement). Describe comparability of assessment methods if there is more than one group	Supplementary	
Bias	9	Describe any efforts to address potential sources of bias	8	
Study size	10	Explain how the study size was arrived at	8	

Continued on next page

Quantitative variables	11	Explain how quantitative variables were handled in the analyses. If applicable, describe which groupings were chosen and why	12,13
Statistical methods	12	(*a*) Describe all statistical methods, including those used to control for confounding	12,13
		(*b*) Describe any methods used to examine subgroups and interactions	12,13
		(*c*) Explain how missing data were addressed	13
		(*d*) *Cohort study*—If applicable, explain how loss to follow-up was addressed<br>*Case-control study*—If applicable, explain how matching of cases and controls was addressed<br>*Cross-sectional study*—If applicable, describe analytical methods taking account of sampling strategy	13
		(*e*) Describe any sensitivity analyses	11
**Results**			
Participants	13*	(a) Report numbers of individuals at each stage of study—eg numbers potentially eligible, examined for eligibility, confirmed eligible, included in the study, completing follow-up, and analysed	14-16
		(b) Give reasons for non-participation at each stage	
		(c) Consider use of a flow diagram	Fig 2
Descriptive data	14*	(a) Give characteristics of study participants (eg demographic, clinical, social) and information on exposures and potential confounders	14-16
		(b) Indicate number of participants with missing data for each variable of interest	14-16
		(c) *Cohort study*—Summarise follow-up time (eg, average and total amount)	14-16
Outcome data	15*	*Cohort study*—Report numbers of outcome events or summary measures over time	16,17
		Case-control study—Report numbers in each exposure category, or summary measures of exposure	
		Cross-sectional study—Report numbers of outcome events or summary measures	
Main results	16	(*a*) Give unadjusted estimates and, if applicable, confounder-adjusted estimates and their precision (eg, 95% confidence interval). Make clear which confounders were adjusted for and why they were included	16,17
		(*b*) Report category boundaries when continuous variables were categorized	16,17
		(*c*) If relevant, consider translating estimates of relative risk into absolute risk for a meaningful time period	n/a

Continued on next page

Other analyses	17	Report other analyses done—eg analyses of subgroups and interactions, and sensitivity analyses	18,19
**Discussion**			
Key results	18	Summarise key results with reference to study objectives	19
Limitations	19	Discuss limitations of the study, taking into account sources of potential bias or imprecision. Discuss both direction and magnitude of any potential bias	22
Interpretation	20	Give a cautious overall interpretation of results considering objectives, limitations, multiplicity of analyses, results from similar studies, and other relevant evidence	20,21
Generalisability	21	Discuss the generalisability (external validity) of the study results	22
**Other information**			
Funding	22	Give the source of funding and the role of the funders for the present study and, if applicable, for the original study on which the present article is based	n/a

*Give information separately for cases and controls in case-control studies and, if applicable, for exposed and unexposed groups in cohort and cross-sectional studies.

Note: An Explanation and Elaboration article discusses each checklist item and gives methodological background and published examples of transparent reporting. The STROBE checklist is best used in conjunction with this article (freely available on the Web sites of PLoS Medicine at http://www.plosmedicine.org/, Annals of Internal Medicine at http://www.annals.org/, and Epidemiology at http://www.epidem.com/). Information on the STROBE Initiative is available at www.strobe-statement.org.
